# Herpes Zoster Vaccine Effectiveness against Incident Herpes Zoster and Post-herpetic Neuralgia in an Older US Population: A Cohort Study

**DOI:** 10.1371/journal.pmed.1001420

**Published:** 2013-04-09

**Authors:** Sinéad M. Langan, Liam Smeeth, David J. Margolis, Sara L. Thomas

**Affiliations:** 1 Department of Epidemiology and Population Health, London School of Hygiene & Tropical Medicine, London, United Kingdom; 2 Department of Dermatology and Center for Clinical Epidemiology and Biostatistics, University of Pennsylvania, Philadelphia, United States of America; University of Geneva Hospitals and Medical School, Switzerland

## Abstract

**Background:**

Herpes zoster is common and has serious consequences, notably post-herpetic neuralgia (PHN). Vaccine efficacy against incident zoster and PHN has been demonstrated in clinical trials, but effectiveness has not been studied in unselected general populations unrestricted by region, full health insurance coverage, or immune status. Our objective was to assess zoster vaccine effectiveness (VE) against incident zoster and PHN in a general population-based setting.

**Methods and Findings:**

A cohort study of 766,330 fully eligible individuals aged ≥65 years was undertaken in a 5% random sample of Medicare who received and did not receive zoster vaccination between 1st January 2007 and 31st December 2009.

Incidence rates and hazard ratios for zoster and PHN were determined in vaccinated and unvaccinated individuals. Analyses were adjusted for age, gender, race, low income, immunosuppression, and important comorbidities associated with zoster, and then stratified by immunosuppression status. Adjusted hazard ratios were estimated using time-updated Cox proportional hazards models.

Vaccine uptake was low (3.9%) particularly among black people (0.3%) and those with evidence of low income (0.6%). 13,112 US Medicare beneficiaries developed incident zoster; the overall zoster incidence rate was 10.0 (9.8–10.2) per 1,000 person-years in the unvaccinated group and 5.4 (95% CI 4.6–6.4) per 1,000 person-years in vaccinees, giving an adjusted VE against incident zoster of 0.48 (95% CI 0.39–0.56). In immunosuppressed individuals, VE against zoster was 0.37 (95% CI 0.06–0.58). VE against PHN was 0.59 (95% CI 0.21–0.79).

**Conclusions:**

Vaccine uptake was low with variation in specific patient groups. In a general population cohort of older individuals, zoster vaccination was associated with reduction in incident zoster, including among those with immunosuppression. Importantly, this study demonstrates that zoster vaccination is associated with a reduction in PHN.

*Please see later in the article for the Editors' Summary*

## Abstract 2

Sinead Marie Langan and colleagues studied a cohort of more than 750,000 individuals over the age of 65 years to assess whether herpes zoster vaccine is effective against incident zoster and post-herpetic neuralgia in an older population.

## Editors' Summary

**Background:**

Chickenpox is an extremely common childhood infectious disease that is caused by the herpes varicella-zoster virus. Children usually recover quickly from chickenpox, but dormant varicella-zoster virus persists throughout life inside the nervous system. The dormant virus causes no symptoms but if it becomes reactivated, it causes shingles (zoster), a painful skin rash. Anyone who has had chickenpox can develop shingles but shingles is most common and most severe in 60–80-year-old people. Indeed, about half of people who live to 85 will have an episode of shingles. Early signs of shingles include burning or shooting pain and tingling or itching. Blister-like sores, which last from 1–14 days, then develop in a region of one side of the body or on one side of the face. The pain of shingles can be debilitating and can continue after the rash disappears—“post-herpetic neuralgia,” which can last for months to years, greatly reduces the quality of life. There is no cure for shingles but early treatment with antivirals may help to prevent lingering pain by inhibiting viral replication.

**Why Was This Study Done?:**

Shingles vaccination can prevent shingles or lessen its effects. In clinical trials, vaccination reduced the incidence of shingles (the proportion of a population who develop shingles in a year) and the incidence of post-herpetic neuralgia, and vaccination against shingles is now recommended in the US for everyone over the age of 60 except individuals with a weakened immune system (for example, people with HIV/AIDS). However, these clinical trials determined the vaccine's efficacy in selected populations under controlled conditions. How effective is the vaccine in unselected populations in routine clinical use? In this cohort study, the researchers assess zoster (shingles) vaccine effectiveness against incident shingles and post-herpetic neuralgia in an unselected population of older individuals in the US. A cohort study follows a group of individuals who differ with respect to specific factors (in this study, vaccination against shingles) to determine how these factors affect the rates of specific outcomes (shingles and post-herpetic neuralgia).

**What Did the Researchers Do and Find?:**

The researchers undertook their cohort study in 766,330 randomly chosen Medicare beneficiaries aged 65 years or more. Medicare is a US government health insurance scheme that mainly helps to pay the health care costs of people aged 65 or older. The researchers used Medicare administrative data to identify which cohort members received zoster vaccination between January 2007 and December 2009 and which developed incident shingles (defined as a first diagnosis of shingles combined with the use of antivirals) or post-herpetic neuralgia (defined as a code for post-herpetic neuralgia, non-specific neuralgia, or a second diagnostic code for shingles 90 days after the first diagnosis combined with a prescription for pain relief, an anticonvulsant, or an antidepressant). Vaccine uptake was low in this unselected study population—only 3.9% of the participants were vaccinated. The vaccination rate was particularly low among black people (0.6% of person-time) and among people with a low income (0.3%). About 13,000 participants developed incident shingles. The shingles incidence rate was 10.0 per 1,000 person-years among unvaccinated participants and 5.4 per 1,000 person-years among vaccinated participants. Vaccine effectiveness against incident shingles was 48%. That is, vaccination reduced the incidence of shingles by 48% (in other words, approximately half as many vaccinated individuals developed shingles as those who were not vaccinated). Vaccine effectiveness against incident shingles among immunosuppressed individuals was lower (37%). Finally, vaccine effectiveness against post-herpetic neuralgia was 59%.

**What Do These Findings Mean?:**

These findings show that shingles vaccine uptake is low among elderly people in the US and varies between different patient groups. They show that shingles vaccination is effective against incident shingles in a general population of older individuals, including those who are immunosuppressed, and suggest that shingles vaccination is effective against post-herpetic neuralgia. However, because these findings rely on administrative data, their accuracy may be affected by misclassification of vaccination and of outcomes. Moreover, because shingles vaccination was not randomized, the vaccinated individuals might have shared other characteristics that were actually responsible for their lower incidence of shingles and/or post-herpetic neuralgia compared to unvaccinated individuals. Despite these limitations, these findings provide useful information for policy makers in countries that are currently considering the introduction of shingles vaccination into routine practice. Moreover, they highlight the need to increase shingles vaccination among elderly individuals in the US, the section of the population at the highest risk of post-herpetic neuralgia.

**Additional Information:**

Please access these Web sites via the online version of this summary at http://dx.doi.org/ 10.1371/journal.pmed.1001420.

- The US Centers for Disease Control and Prevention have detailed information about all aspects of shingles (zoster), including information on vaccination for the public and for health care professionals, and a personal story about shingles
- The NIH Senior Health website includes information on shingles and a video describing a personal experience of shingles
- The UK National Health Service Choices also provides information about all aspects of shingles and a personal story
- MedlinePlus provides links to other resources about shingles (in English and Spanish)
- The British Association of Dermatologists website has an information leaflet on shingles
- The New Zealand Dermatological Society website has a leaflet on shingles

## Introduction

Herpes zoster is a significant public health problem affecting 1 million individuals in the US per year and associated with important sequelae [1],[2]. Herpes zoster occurs following reactivation of latent varicella zoster virus (VZV) infection and presents with a painful vesicular rash, which frequently in older individuals leads to prolonged pain, post-herpetic neuralgia (PHN), with a major impact on quality of life [2]. Vaccine efficacy has been shown in trials [3],[4]; in a selected insured population [5]; and among people with any of five specific immune-mediated diseases [6] but not among an unselected population in a clinical setting. Zhang et al. demonstrated that despite Advisory Committee for Immunization Practices (ACIP) recommendations, individuals with immunosuppression received the live herpes zoster vaccine in clinical practice [7]. The lack of adherence to ACIP recommendations on vaccination is not entirely surprising given that individuals with immunosuppression are not only at increased risk of incident herpes zoster but also at significantly increased risk of herpes zoster complications, in particular prolonged, severe PHN [8],[9]. Previous research has suggested that the varicella vaccine may be efficacious and safe in people with immunosuppressive disorders [10]–[12]. Similar evidence about vaccine effectiveness (VE) is lacking in relation to the zoster vaccine in individuals with serious immune suppression, beyond effectiveness among those with the selected immune-mediated disorders examined to date.

Important outstanding research questions with great relevance to policy include VE in unselected population-based elderly US populations; this includes effectiveness against PHN, which has not been assessed in routine practice. The report by Zhang et al. also highlights the additional importance of studying further VE in those with immunosuppression [6]. This is the first study to the best of our knowledge to assess the effectiveness of herpes zoster vaccine against both incident herpes zoster and PHN in an unselected older population including those with immunosuppression.

## Methods

### Ethics

Ethics approval was obtained from Centers for Medicare & Medicaid Services (CMS) (data use agreement 21520) and the Ethics committee of the London School of Hygiene and Tropical Medicine. Any data cell containing fewer than 11 beneficiaries have not been shown as per the CMS Data Use Agreement.

### Data Source

Medicare is a US administrative claims program mainly for individuals aged >65 y covering 15% of the US population. There are 44 million beneficiaries, of which more than half the individuals are aged 65–75 y.[13] This study was based on the 5% random Medicare Standard Analytic Files (SAF) including Denominator, Inpatient hospital discharge records (MedPAR), Physician/Supplier (Carrier) and Outpatient files from January 1^st^ 2007 to December 31^st^ 2009 obtained from the CMS.

### Study Population

**Figure 1:**
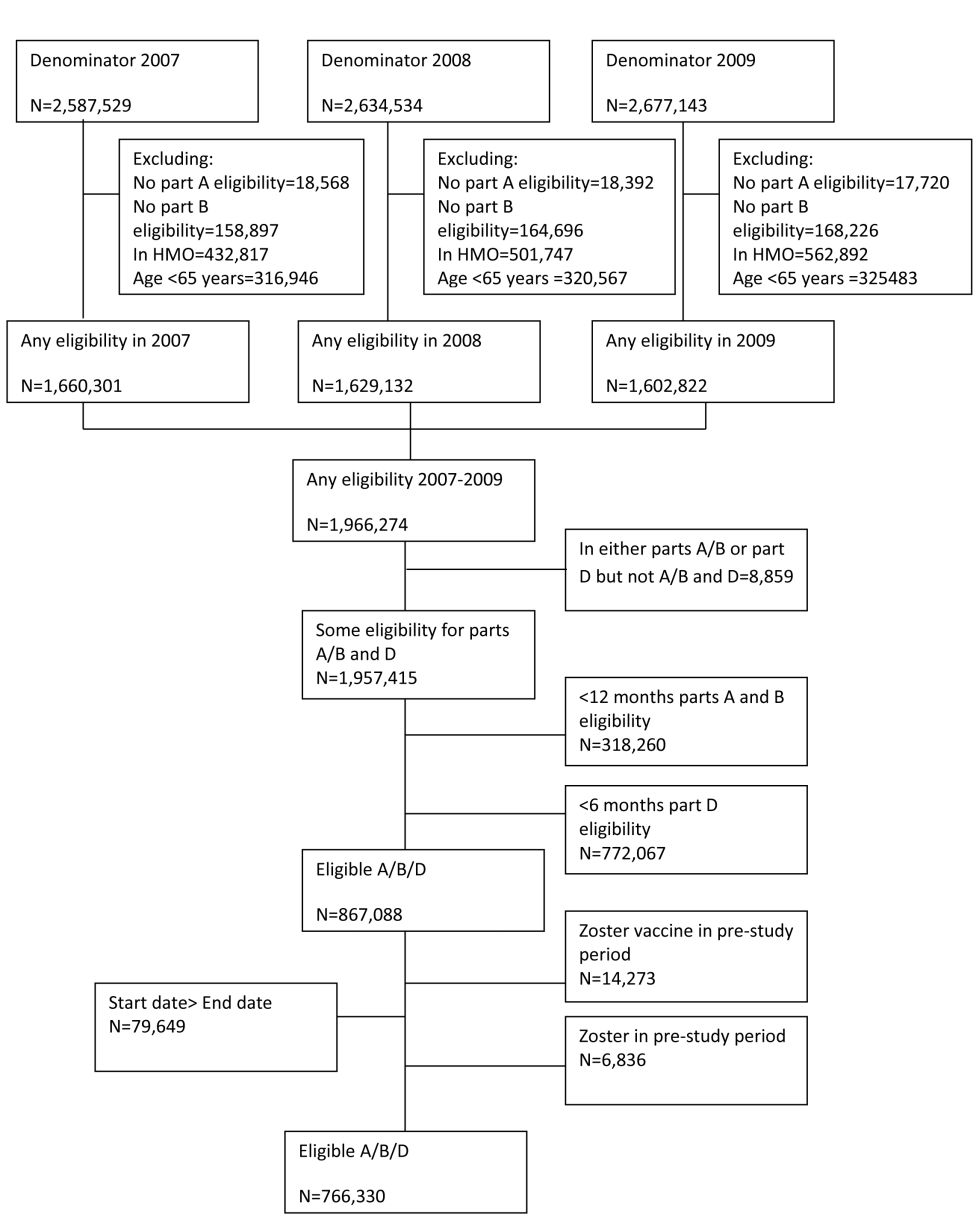
Flow chart of analysis cohort.

Study participants were aged 65 y or greater with at least 12 mo continuous enrolment in Medicare parts A (which covers inpatient care) and B (physician services and facility charges) and at least 6 mo continuous enrolment in part D (drug benefits) of Medicare. The start of follow-up was the first date an individual fulfilled all the eligibility criteria with an additional 12-mo baseline pre-study observation period added to ensure observation of incident rather than prevalent zoster. End of follow-up was defined as the earliest of end of eligibility, date of death, development of herpes zoster, or the end of the study period. Individuals enrolled in health maintenance organizations or Medicare Advantage plans were excluded from the study as their records are not processed by CMS, hence information on clinical events is not available. Individuals with episodes of herpes zoster in the first year pre-study observation period were excluded from analysis to exclude prevalent cases. Additionally, individuals who received the herpes zoster vaccine during the baseline pre-study observation period were excluded from analysis (Figure 1).

### Exposure

Herpes zoster vaccine was identified based on the presence of Current Procedural Terminology (CPT) code 90736. Additionally, specific National Drug Codes (NDCs) for herpes zoster vaccine were identified. A definite administration date was considered present if a CPT code 90741 or Healthcare Common Procedural Coding system (HCPCS) code G0377 was present within 7 d of vaccine purchase; otherwise the date of recording of the NDC code for herpes zoster vaccine was considered to be the administration date.

### Outcomes

Incident herpes zoster cases were identified as those with both the presence of a specific International Classification of Diseases, Ninth Revision, Clinical Modification (ICD-9-CM) diagnostic code for herpes zoster, excluding those with specific ICD-9-CM codes for PHN, and the use of antivirals, including acyclovir, famciclovir, or valacyclovir, within 7 d either before or after the diagnostic code for herpes zoster [6]. Cases were identified from outpatient, inpatient, or health care provider (carrier) files. The approach of using both the presence of diagnostic codes and receipt of antivirals was proposed by Zhang et al. to increase the positive predictive value of a herpes zoster diagnosis in an administrative data source [6]. Incident herpes zoster was defined as an episode of herpes zoster without any evidence of herpes zoster or PHN for at least 1 y previously. A sensitivity analysis was undertaken defining incident herpes zoster as the presence of an ICD-9 code for herpes zoster irrespective of receipt of antiviral therapy. PHN was identified using a modification of the method proposed by Klompas et al. for administrative sources [14]. On the basis of this method, PHN was identified as those with a first episode of zoster with a further zoster diagnostic code after 90 d with a relevant prescription for analgesia, anticonvulsant, or antidepressant therapy on the same day as the recorded consultation. The presence of codes for non-specific neuralgia or for neurological complications of zoster after 90 d was also consistent with PHN. The PHN analysis was repeated after 30 d using the same diagnostic criteria.

### Covariates

Current age was determined using date of birth identified from the Medicare beneficiary file; age was categorized into 5-y age bands as a time-varying covariate. Age at the start of the study was also modelled as a continuous variable in a sensitivity analysis. Race was identified from the Medicare SAF Denominator files, derived from the Social Security Administration's master beneficiary record (designated via self-report) and categorized into white, black, and other (not including those with missing data on race). It is critical to study race in any study of zoster epidemiology as incidence rates in individuals with black skin are significantly lower than in white individuals [15]. “State buy -in” at any point during follow-up was assessed as a marker for low income. “State buy-in” reflects that the state pays Medicare premiums for an individual who is eligible due to low income. It can be assumed that people with state buy-in have resources that are less than twice that of the Supplemental Security Income threshold; hence “state buy-in” can be used a proxy for low income. We also determined the proportions of individuals receiving herpes zoster vaccination and developing incident herpes zoster by state and used these data to define quintiles of states defined by each of these two variables.

Immunosuppression status was identified as a time-varying covariate. Once individuals developed leukaemia, lymphoma, or HIV, as determined by the presence of two diagnostic ICD-9-CM codes on different days within outpatient, inpatient, or provider files, they were defined as being immunosuppressed from that point forward. These specific disorders were selected as these are specific ACIP contraindications for the zoster vaccine [7].

Immunosuppressive medications were identified from the part D drug files and an individual was defined as being immunosuppressed for 6 mo following the prescription of any immunosuppressive medication. If a patient received a further script during that period, they remained immunosuppressed. Other comorbidities including immune-mediated disorders or others previously identified as being associated with increased risks of zoster such as chronic obstructive pulmonary disease (COPD), diabetes, and systemic lupus erythematosis (SLE) were identified from medical records by the presence of two ICD-9-CM codes on different days in the outpatient or carrier (provider) files or one or more codes from inpatient records. Individuals with autoimmune disorders such as SLE were considered immunocompetent unless they received immunosuppressive therapy; these disorders are not ACIP contraindications to vaccination.

### Statistical Analysis

Characteristics associated with receipt of the herpes zoster vaccine were explored by examining proportions of person-years of follow-up contributed by demographic and clinical attributes. Incidence rates for herpes zoster and PHN overall and by population characteristics were determined by identifying the number of events divided by person-years of follow-up. Cox regression was used to derive hazard ratios for herpes zoster and PHN in the vaccinated compared with the unvaccinated, adjusting for relevant confounders identified from the previous literature [3],[15], including age, gender, race, low income, immunosuppression, and comorbidities associated with herpes zoster, with age and immunosuppression being included in the analysis as time-varying covariates. Other comorbidities including COPD were treated as binary variables. State quintiles of vaccination and zoster incidence were added to the model to determine if they were confounders. Checks for collinearity were undertaken by assessing variance inflation factors for independent variables. Interaction terms were explored for associations between vaccination and age group and gender. VE was calculated as (1 – the adjusted hazard ratio). Stratified analysis of VE by immune status was adjusted for demographic characteristics with a sensitivity analysis adjusting for other comorbidities. A further sensitivity analysis was undertaken assessing VE against PHN using logistic regression amongst those with zoster with at least 6 mo of follow-up following zoster. All analyses were undertaken using STATA (version 11.0).

## Results

### Vaccination Rates

Of the 766,330 eligible participants, 29,785 (3.9% of people; 2.1% of person-time) had herpes zoster vaccination during the study period. Vaccination rates were lower in the oldest age group (1.5% in those aged 80 y or greater), in black individuals (0.3% compared to 2.4% in white individuals), and lower in those with evidence of low income (Table 1)—0.6% in those with evidence of low income were vaccinated as compared to 2.6% in individuals with no evidence of low income. 140,925 individuals were immunosuppressed at some point during follow-up and 4,469 of these individuals were immunosuppressed at the time of herpes zoster vaccination.

**Table 1:** Person-years by vaccination status and characteristics.

Characteristic	Person-Years (*n*)^a^	Percent Vaccinated 28,291 Person-Years (*n*)	Percent Unvaccinated 1,291,832 Person-Years (*n*)
**Age (y)** b			
65–69	273,312 (202,703)	2.5 (8,783)	97.5 (193,920)
70–74	302,422 (160,833)	2.6 (8,039)	97.4 (152,794)
75–79	262,110 (142,496)	2.4 (6,154)	97.6 (136,342)
≥80	482,278 (260,255)	1.5 (6,903)	98.5 (253,352)
**Gender**			
Male	420,652 (247,941)	2.0 (9,148)	98.0 (238,793)
Female	899,469 (518,391)	2.2 (20,775)	97.8 (497,616)
**Race** c			
Black	111,633 (66,506)	0.3 (390)	99.7 (66,119)
White	1,119,377 (646,805)	2.4 (28,016)	97.6 (618,789)
Other	87,562 (52,046)	1.6 (1,484)	98.4 (50,562)
**Low income**			
No	990,649 (570,182)	2.6 (27,523)	97.4 (542,659)
Yes	329,472 (196,150)	0.6 (2,400)	99.4 (193,750)
**COPD**			
No	960,243 (553,697)	2.4 (24,696)	97.6 (529,001)
Yes	359,878 (212,635)	1.4 (5,277)	98.6 (207,408)
**IBD**			
No	1,298,483 (753,863)	2.1 (29,376)	97.9 (724,489)
Yes	21,638 (12,467)	2.5 (547)	97.5 (11,920)
**Kidney disease**			
No	1,013,681 (582,568)	2.3 (25,370)	97.7 (557,198)
Yes	306,440 (183,764)	1.4 (4,553)	98.6 (179,211)
**Diabetes mellitus**			
No	802,500 (466,316)	2.5 (21,111)	97.5 (445,205)
Yes	517,621 (300,016)	1.6 (8,812)	98.4 (291,204)
**RA**			
No	1,249,788 (726,516)	2.2 (28,727)	97.8 (697,789)
Yes	70,333 (39,816)	1.6 (1,196)	98.4 (38,620)
**SLE**			
No	1,311,411 (761,406)	2.1 (29,745)	97.9 (731,661)
Yes	8,710 (4,926)	2.0 (178)	98.0 (4,748)
**Immune suppression** d			
No	1,233,333 (625,409)	2.1 (24,392)	97.9 (601,017)
Yes	86,788 (140,923)	2.3 (5,531)	97.7 (135,392)

a Individuals could contribute person-time to more than one category.

b For determination of numbers, age at vaccination used if vaccinated; otherwise baseline age.

c Missing race information for 975 people (0.1%).

d At any stage during the study.

IBD, inflammatory bowel disease; RA, rheumatoid arthritis.

### Herpes zoster Incidence Rates

Incidence rates for herpes zoster using the antiviral definition were higher in older age groups, in women, in those with any immunosuppression (adjusted hazard ratio 1.80 [95% CI 1.70–1.90]) and in those with specified immune-mediated disorders, including inflammatory bowel disease and SLE, and other disorders such as chronic kidney disease and COPD (Table 2). Lower incidence rates were seen in people who reported being black (adjusted hazard ratio 0.51 ([95% CI 0.47–0.56]) and those with any evidence of low income (adjusted hazard ratio 0.86 [95% CI 0.82–0.90]).

**Table 2:** Incidence rates for herpes zoster by disease definition and patient characteristics.

Characteristic	Antiviral Definition					General Definition				
	Herpes zoster Cases	Person-Years (1,000)	Incidence Rate per 1,000 Person-Years (95% CI)	Crude Hazard Ratio (95% CI)	Adj. Hazard Ratio (95% CI)^a^	Herpes zoster Cases	Person-Years (1,000)	Incidence Rate per 1,000 Person-Years (95% CI)	Crude Hazard Ratio (95% CI)	Adj. Hazard Ratio (95% CI)
**Overall**	13,112	1,320.1	9.9 (9.8–10.1)	—	—	19,722	1,314.8	15.0 (14.8–15.2)	—	—
**Age (y)**										
65–69	2,350	273.3	8.6 (8.2–8.9)	1.0	1.0	3,433	272.5	12.6 (12.2–13.0)	1.0	1.0
70–74	2,921	302.4	9.6 (9.3–10.0)	1.11 (1.05–1.17)	1.09 (1.03–1.15)	4,288	301.3	14.2 (13.8–14.7)	1.13 (1.08–1.18)	1.10 (1.06–1.16)
75–79	2,759	262.1	10.5 (10.1–10.9)	1.21 (1.15–1.28)	1.15 (1.09–1.21)	4,100	261.0	15.7 (15.2–16.2)	1.25 (1.19–1.30)	1.17 (1.12–1.23)
≥80	5,082	482.3	10.5 (10.2–10.8)	1.21 (1.15–1.27)	1.11 (1.06–1.17)	7,901	479.9	16.4 (16.1–16.8)	1.31 (1.26–1.36)	1.19 (1.14–1.24)
**Gender**										
Male	3,470	420.6	8.2 (8.0–8.5)	1.0	1.0	5,182	419.2	12.4 (12.0–12.7)	1.0	1.0
Female	9,642	899.5	10.7 (10.5–10.9)	1.30 (1.25–1.35)	1.30 (1.25–1.35)	14,540	895.5	16.2 (16.0–16.5)	1.31 (1.27–1.35)	1.31 (1.27–1.35)
**Race**										
White	11,762	1,119.4	10.5 (10.3–10.7)	1.0	1.0	17,631	1,114.6	15.8 (15.6–16.0)	1.0	1.0
Black	591	111.6	5.3 (4.9–5.7)	0.50 (0.46–0.55)	0.51 (0.47–0.56)	940	111.3	8.4 (7.9–9.0)	0.53 (0.50–0.57)	0.55 (0.51–0.59)
Other	749	87.6	8.5 (8.0–9.2)	0.81 (0.76–0.88)	0.89 (0.83–0.96)	1,129	87.2	12.9 (12.2–13.7)	0.90 (0.59–1.37)	0.91 (0.85–0.97)
**Low income**										
No	10,242	991.0	10.3 (10.1–10.5)	1.0	1.0	15,432	986.4	15.6 (15.4–15.9)	1.0	1.0
Yes	2,870	329.5	8.7 (8.4–9.0)	0.84 (0.81–0.88)	0.86 (0.82–0.90)	4,290	328.4	13.1 (12.7–13.5)	0.83 (0.81–0.86)	0.84 (0.81–0.87)
**COPD**										
No	9,014	960.2	9.4 (9.2–9.6)	1.0	1.0	13,388	956.4	14.0 (13.8–14.2)	1.0	1.0
Yes	4,098	360.0	11.4 (11.0–11.7)	1.21 (1.17–1.26)	1.13 (1.09–1.17)	6,334	358.3	17.7 (17.2–18.1)	1.26 (1.22–1.30)	1.17 (1.13–1.20)
**IBD**										
No	12,811	1,298.5	9.9 (9.7–10.0)	1.0	1.0	19,262	1,293.2	14.9 (14.7–15.1)	1.0	1.0
Yes	301	21.6	13.9 (12.4–15.6)	1.41 (1.26–1.58)	1.25 (1.11–1.40)	460	21.5	21.4 (19.5–23.4)	1.44 (1.31–1.57)	1.26 (1.15–1.38)
**Kidney disease**										
No	9,646	1,013.7	9.5 (9.3–9.7)	1.0	1.0	14,258	1,009.8	14.1 (13.9–14.3)	1.0	1.0
Yes	3,466	306.4	11.3 (10.9–11.7)	1.19 (1.14–1.24)	1.16 (1.11–1.21)	5,464	305.0	17.9 (17.4–18.4)	1.27 (1.23–1.31)	1.22 (1.18–1.26)
**Diabetes Mellitus**										
No	7,885	802.5	9.8 (9.6–10.0)	1.0	1.0	11,734	799.3	14.7 (14.4–14.9)	1.0	1.0
Yes	5,227	517.6	10.1 (9.8–10.4)	1.03 (0.99–1.06)	1.03 (0.99–1.07)	7,988	515.4	15.5 (15.2–15.8)	1.05 (1.02–1.08)	1.05 (1.02–1.08)
**RA**										
No	12,178	1,249.8	9.7 (9.6–9.9)	1.0	1.0	18,274	1,244.7	14.7 (14.5–14.9)	1.0	1.0
Yes	934	70.3	13.23(12.4–14.1)	1.36 (1.27–1.45)	1.15 (1.07–1.23)	1,448	70.0	20.7 (19.6–21.8)	1.41 (1.33–1.48)	1.19 (1.12–1.25)
**SLE**										
No	12,980	1,311.4	9.9 (9.7–10.1)	1.0	1.0	19,514	1,306.1	14.9 (14.7–15.1)	1.0	1.0
Yes	132	8.7	15.1 (12.8–18.0)	1.53 (1.29–1.82)	1.24 (1.04–1.47)	208	8.7	24.0 (20.9–27.5)	1.61 (1.40–1.84)	1.29 (1.12–1.48)
Immune suppression										
No	11,528	1,233.3	9.3 (9.2–9.5)	1.0	1.0	17,411	1,228.1	14.2 (14.0–14.4)	1.0	1.0
Yes	1,584	86.8	18.2 (17.4–19.2)	1.94 (1.84–2.05)	1.80 (1.70–1.90)	2,311	86.6	26.7 (25.6–27.8)	1.89 (1.80–1.97)	1.72 (1.64–1.80)
**Vaccinated**										
No	12,958	1,291.8	10.0 (9.8–10.2)	1.0	1.0	193,897	1,287.0	15.1 (14.9–15.3)	1.0	1.0
Yes	154	28.3	5.4 (4.6–6.4)	0.55 (0.47–0.64)	0.52 (0.44–0.61)	325	27.8	11.7 (10.5–13.0)	0.77 (0.69–0.86)	0.76 (0.68–0.85)

a Adjusted for age, gender, race, immunosuppression status, low income, COPD, IBD (inflammatory bowel disease), kidney disease, diabetes mellitus, RA (rheumatoid arthritis), and SLE,

### Herpes zoster Vaccine Effectiveness

**Figure 2:**
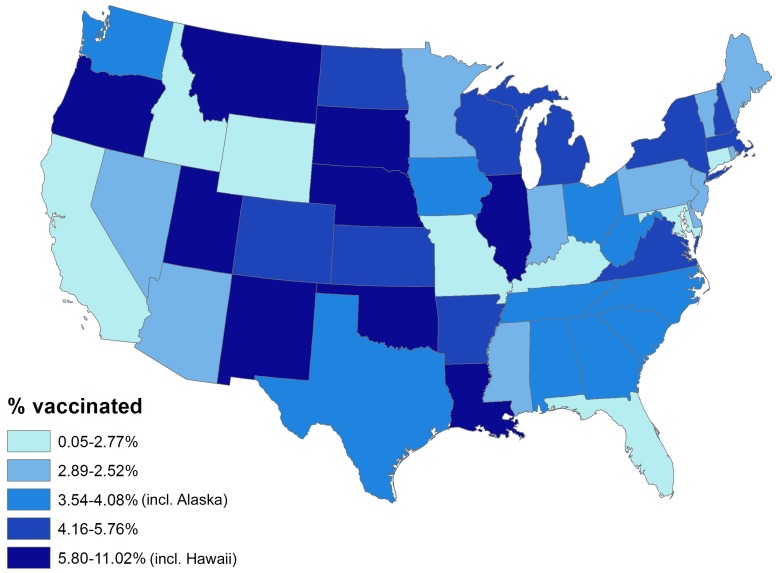
Percentage vaccinated by state. Alaska and Hawaii not included in this figure for graphical reasons.

Overall, 154 vaccinees experienced incident herpes zoster episodes (defined using the specific antiviral definition) during 28,291 person-years of follow-up compared to 12,958 events in 1,291,829 person-years of follow-up in those not vaccinated, giving an incidence rate of herpes zoster in vaccinees of 5.4 (95% CI 4.6–6.4) per 1,000 person-years compared to 10.0 (95% CI 9.8–10.2) per 1,000 person-years in those not vaccinated. All variance inflation factors were less than 1.2, suggesting collinearity was not a major issue, and no significant interactions were detected. The overall vaccine effectiveness (VE) for herpes zoster in vaccinees adjusted for age, gender, race, immunosuppression, low income, and comorbidity was 0.48 (95% CI 0.39–0.56) (Table 3). Incorporating age at the start of the study as a continuous variable did not change study findings: adjusted VE, 0.48 (95% CI 0.40–0.56). The median time to vaccine failure was 168 d. In immunocompromised vaccinees, there were 24 events in 1,981 person-years of follow-up, giving an adjusted VE of 0.37 (95% CI 0.06–0.58). Adjusting for state quintiles of either proportions receiving herpes zoster vaccination or proportions with incident herpes zoster did not modify study findings (adjusted VE 0.48 [95% CI 0.30–0.56] and 0.48 [95% CI 0.30–0.56, respectively]). Proportions vaccinated per state varied from 0.05% to 11.02% (Figure 2) and proportions of individuals developing incident herpes zoster during follow-up per state varied from 0% to 7%. At 90 d or greater following zoster, the adjusted VE was 0.59 (95% CI 0.21–0.79) for PHN in vaccinees compared to those not vaccinated, after adjusting for age, gender, race, and other comorbidities (numbers suppressed to remain compliant with CMS's small-sized cell privacy policy). At 30 d or longer following zoster, 16 vaccinees developed PHN during 71,457 person-years of follow-up compared to 1,665 events during 2,563,404 person-years of follow-up in those not vaccinated, giving an adjusted VE of 0.62 (95% CI 0.37–0.77) for PHN, after adjusting for age, gender, race, immunosuppression status, and other comorbidities (Table 4). Results of the logistic regression analysis amongst those with zoster showed materially similar estimates of protection against PHN, albeit with wider confidence intervals (adjusted VE 0.64 [95% CI 0.11–0.85]). Lower VE against incident herpes zoster and PHN was seen when using the general rather than the specific disease definition.

**Table 3:** Zoster vaccine effectiveness against incident herpes zoster by characteristics and disease definition.

Vaccination Status	Antiviral Definition^a^					General Definition				
	Events	Person-Years (1,000)	Incidence Rate per 1,000 PY (95% CI)	Crude Hazard Ratio (95% CI)	Adj. Hazard Ratio* (95% CI)^b^	Events	Person-Rears (1,000)	Incidence Rate per 1,000 PY (95% CI)	Crude Hazard Ratio (95% CI)	Adj. Hazard Ratio (95% CI)^b^
Unvaccinated	12,958	1,291.8	10.0 (9.8–10.2)	1.0	1.0	19,385	1,286.9	15.1 (14.9–15.3)	1.0	1.0
Vaccinated overall	154	28.3	5.4 (4.6–6.4)	0.55 (0.47–0.64)	0.52 (0.44–0.61)	325	27.8	11.7 (10.5–13.0)	0.79(0.71–0.88)	0.76 (0.68–0.85)
Unvaccinated immunocompetent	11,398	1,207.0	9.4 (9.3–9.6)	1.0	1.0	17,110	1,202.3	14.2 (14.0–14.4)	1.0	1.0
Vaccinated immunocompetent	130	26.3	4.9 (4.2–5.9)	0.53 (0.44–0.63)	0.49 (0.41–0.59)	289	25.8	11.2 (10.0–12.6)	0.80 (0.71–0.90)	0.75 (0.67–0.84)
Unvaccinated immunosuppressed^c^	1,560	85.0	18.3 (17.5–19.3)	1.0	1.0	2,275	84.6	26.9 (25.8–28.0)	1.0	1.0
Vaccinated immunosuppressed^c^	24	2.0	12.1 (8.1–18.1)	0.65 (0.44–0.98)	0.63 (0.42–0.94)	36	2.0	18.2 (13.2–25.3)	0.68 (0.49–0.94)	0.66 (0.47–0.91)

a Definition requiring antiviral administration within 7 d before or after the diagnostic code for herpes zoster.

b Adjusted for age, gender, race, low income, COPD, IBD, kidney disease, diabetes mellitus, rheumatoid arthritis, and SLE. Overall estimates were also adjusted for immunosuppression status. Stratified analysis by immune status adjusted for age, gender, race, and low income.

c Immunosuppression defined as individuals with leukaemia, lymphoma, and HIV or during and for 6 mo after a prescription for an immunosuppressive drug including oral corticosteroids.

**Table 4:** Zoster vaccine effectiveness against PHN by characteristics and disease definition.

Timing of Outcome	Vaccination Status	Antiviral Definition^a^					General Definition				
		Events	Person-Years (1,000)	Incidence Rate per 1,000 Person-Years (95% CI)	Crude Hazard Ratio (95% CI)	Adj. Hazard Ratio (95% CI)^b^	Events	Person-Years (1,000)	Incidence Rate per 1,000 PY (95% CI)	Crude Hazard Ratio (95% CI)	Adj. Hazard Ratio (95% CI)^b^
**PHN 30**	**Unvaccinated**	1,665	2,563.4	0.65 (0.62–0.68)	1.0	1.0	2,241	2,507.0	0.89 (0.86–0.93)	1.0	1.0
	**Vaccinated**	16	71.4	0.22 (0.14–0.36)	0.39 (0.24–0.64)	0.38 (0.23–0.63)	29	70.1	0.41 (0.29–0.59)	0.52 (0.36–0.76)	0.52 (0.36–0.75)
**PHN 90**	**Unvaccinated**	871	2,616.5	0.33 (0.31–0.35)	1.0	1.0	1,220	2,581.0	0.47 (0.45–0.50)	1.0	1.0
	**Vaccinated**	c	c	c	0.42 (0.22–0.81)	0.41 (0.21–0.79)	19	70.9	0.27 (0.17–0.42)	0.64 (0.40–1.00)	0.62 (0.39–0.97)

a Definition requiring antiviral administration within 7 d before or after the diagnostic code for herpes zoster.

b Adjusted for age, gender, race, immunosuppression status, low income, COPD, IBD, kidney disease, diabetes mellitus, rheumatoid arthritis, and SLE.

c Numbers suppressed to remain compliant with CMS's small-sized cell privacy policy.

## Discussion

This is the first population-based study, to the best of our knowledge, to demonstrate the effectiveness of herpes zoster vaccination against PHN in a routine population setting. Study findings are consistent with efficacy data from the Shingles Prevention Study (SPS) randomised controlled trial (RCT) [3]. This work complements the findings of previous observational studies that have shown effectiveness of herpes zoster vaccination in immunocompetent insured individuals in southern California and in older individuals with selected immune-mediated diseases, as this study is the first study to the best of our knowledge to determine effectiveness against incident herpes zoster in a population-based cohort of older individuals across the US, not restricted by geographic region, immune status, or insurance status [5],[6].

Low uptake of herpes zoster vaccination (4%) was seen overall with variations in uptake by age, race, and low income levels. Overall VE of 48% was demonstrated against incident herpes zoster, 62% against PHN after 30 d and 59% against PHN after 90 d. In immunosuppressed individuals, VE against incident herpes zoster was 37%.

In the SPS RCT, Oxman et al. demonstrated herpes zoster vaccine efficacy against incident herpes zoster (51%) and PHN (67%) in 38,546 immunocompetent individuals (19,270 of whom were vaccinated) aged 60 y or greater with no history of herpes zoster [3]. Subsequent to this study, Schmader et al. performed an RCT in 22,439 immunocompetent individuals aged 50–59 y in North America and Europe, demonstrating vaccine efficacy of 69.8% for incident herpes zoster; efficacy against PHN was not determined [4]. Our findings are closer to those of the SPS study with similar estimates and confidence intervals for VE against incident zoster, which likely relates to the older age of participants in our study population. Our findings for VE against PHN were similar to those observed in the SPS trial [3]. RCTs typically have excellent internal validity, but post-licensure observational studies are necessary to inform generalisability of research findings.

Our study also confirms the results of the study by Tseng et al., which demonstrated the effectiveness of the herpes zoster vaccine against herpes zoster incidence in 75,761 immunocompetent vaccinees aged 60 y or greater matched (1∶3) to unvaccinated members; all study participants were fully insured individuals in Kaiser Permanente Southern California [5]. The authors reported VE of 55% against incident herpes zoster, which is very similar to our estimates, despite differences in the study population. Zhang et al. assessed herpes zoster VE in 463,541 Medicare beneficiaries aged 60 y or greater with a restricted range of immune-mediated diseases and 18,683 vaccinees and found an adjusted hazard ratio of 0.61 (95% CI 0.52–0.71) overall [6]. The lower VE in this population likely reflects their underlying immunosuppression. The authors did not observe an increase in risk of herpes zoster following vaccination in those with immune-mediated disease, nor did they detect any cases of herpes zoster in individuals vaccinated while on biologic therapy.

Medicare is an administrative data source so some misclassification of exposures and outcomes is possible. However, this misclassification is likely to be random leading to the possibility of bias towards the null. Despite taking steps to guard against misclassification of the PHN by using the method proposed by Klompas et al. [14] for administrative data sources, the incidence of PHN in this study is lower than in the SPS [3]—1.38 per 1,000 person-years in the placebo group compared to 0.65 per 1,000 in the unvaccinated in our study—which might suggest some misclassification. In a recent study combining administrative data with medical record review, 3.9% of those with zoster developed PHN, which is lower than the 6.7% of people observed in this study [16]. There is a possibility that we underestimated herpes zoster vaccine uptake if individuals paid for their own vaccination; however, given that all of these individuals have part D Medicare (drug benefit) coverage, individual payment is unlikely because of the cost of the vaccine (US$159 for a single dose, not including administration costs). As these are observational data, the exposure—herpes zoster vaccination—was not randomly allocated. Our study demonstrates that vaccine uptake was not random and was likely to have been influenced by the demographic characteristics of beneficiaries. As data on exposures and outcomes were not collected for research purposes, there are unmeasured potential confounders including smoking and obesity, which are not routinely recorded in an administrative data source, despite the availability of diagnostic codes. Previous studies have not suggested that either of these covariates are major risk factors for the development of incident herpes zoster or PHN and, therefore, they are unlikely to confound the associations [15]. The study period was relatively short; the first 12 mo following eligibility was excluded to enable study of incident rather than prevalent herpes zoster. This limited duration results in the inability to study long-term vaccine effects but does not impact the study of VE. Additionally, while VE was assessed in individuals with immunosuppression, assessment of adverse effects or vaccine safety was not the hypothesis under study. The number of vaccinated immunosuppressed individuals in the study was modest resulting in a lack of precision of the estimate of VE in this group.

Our study is a large population-based cohort; the size of the cohort gives sufficient statistical power to study VE against herpes zoster and PHN and results are estimated relatively precisely; our findings would be unlikely if there was no effect in the population. In addition, Medicare beneficiaries are reasonably representative of the general US elderly population, with 98% of Americans aged 65 y or greater being enrolled in Medicare in 2009, increasing the generalisability of our findings [13]. Medicare datasets have high quality data available on demographic details of beneficiaries and clinical encounters, including prescription data. A strict definition for herpes zoster was used and therefore misclassification of incident herpes zoster is not likely, although it is not possible to completely exclude misclassification [16],[17]. The higher VE when using the specific definition could reflect some misclassification of zoster using the general definition. Alternatively, those with zoster who did not receive antivirals might include a large proportion with very mild disease; in the SPS, the zoster vaccine was shown to have higher efficacy against zoster with appreciable acute morbidity than against any zoster [3]. If those not receiving antivirals had milder incident zoster, this could lead to over-estimation of VE while providing reasonable estimates for significant zoster episodes. In this study VE was determined after adjusting for a wide range of confounders, including demographic details, immunosuppression, and immune-mediated diseases; despite the large size of this dataset, in some instances adjusting for confounding led to wide confidence intervals, for example when assessing VE in immunocompromised vaccinees.

### Conclusions

Herpes zoster vaccination was associated with a significant reduction in incident herpes zoster and PHN in routine clinical use. This study also supports effectiveness of the vaccine against incident herpes zoster in immunosuppressed individuals, although the number of immunosuppressed individuals was small, resulting in lack of precision in the estimate. Given that these individuals are at greatest risk of both herpes zoster and complications, this may have important implications for policy. The findings are relevant beyond US medical practice, being of major importance to the many countries, including the UK, that are actively considering introducing the zoster vaccine into routine practice in the near future.

Despite strong evidence supporting its effectiveness, clinical use remains disappointingly low with particularly low vaccination rates in particular patient groups. This study shows that herpes zoster vaccination is associated with a reduction in PHN in routine clinical use. As PHN is the major complication of herpes zoster and is associated with highly significant morbidity and adverse impacts on quality of life, substantial efforts are needed to increase vaccine use in routine care of elderly individuals.

## Abbreviations

ACIP: Advisory Committee for Immunization Practices
CMS: Centers for Medicare & Medicaid Services
COPD: chronic obstructive pulmonary disease
PHN: post-herpetic neuralgia
RCT: randomised controlled trial
SLE: systemic lupus erythematosis
SPS: Shingles Prevention Study
VE: vaccine effectiveness
